# Rationale and methods of the multicenter randomised trial of a heart failure management programme among geriatric patients (HF-Geriatrics)

**DOI:** 10.1186/1471-2458-11-627

**Published:** 2011-08-05

**Authors:** Carlos Rodríguez Pascual, Emilio Paredes Galán, Jose Luis Gonzalez Guerrero, Rocio Menendez Colino, Pedro Abizanda Soler, Mercedes Hornillos Calvo, Juan Jose Solano Jaurieta, Jorge Manzarbeitia Arambarri, Jose Manuel Ribera Casado, Fernando Rodríguez-Artalejo

**Affiliations:** 1 Servicio de Geriatría. Hospital Meixoeiro. Complejo Hospitalario Universitario de Vigo. Departamento de Medicina. Universidad de Santiago de Compostela. Meixoeiro s.n. 36200 Vigo (Pontevedra). SPAIN; 2 Departamento de Medicina Preventiva y Salud Pública. Universidad Autónoma de Madrid/IdiPaz. CIBER of Epidemiology and Public Health. C/Arzobispo Morzillo s.n. 28029 Madrid. SPAIN; 3 Servicio de Cardiología. Hospital Meixoeiro. Complejo Hospitalario Universitario de Vigo. Meixoeiro s.n. 36200 Vigo (Pontevedra). SPAIN; 4 Servicio de Geriatría. Hospital Nuestra Señora de la Montaña. Complejo Hospitalario de Caceres. Av. España no 2. 10004 Caceres. SPAIN; 5 Servicio de Geriatría. Hospital Universitario La Paz. Departamento de Medicina. Facultad de Medicina. Universidad Autónoma de Madrid. Po de la Castellana, 261. 28046 Madrid. SPAIN; 6 Servicio de Geriatría. Complejo Hospitalario de Albacete. Departamento de Medicina. Universidad de Castilla-La Mancha. C/Seminario 4. 02006 Albacete. SPAIN; 7 Servicio de Geriatría. Hospital Universitario de Guadalajara. Departamento de Medicina, Universidad de Alcalá. C/Donantes de Sangre s.n.19002 Guadalajara. SPAIN; 8 Servicio de Geriatría. Hospital Monte Naranco. Complejo Hospitalario de Oviedo. Departamento de Medicina, Universidad de Oviedo. Avda. Dres. Fernandez Vega s/n33012 Oviedo. SPAIN; 9 Servicio de Geriatría. Hospital Universitario de Getafe. Departamento de Medicina. Universidad Europea de Madrid. Ctra. de Toledo, Km. 12,5. 28905 Getafe (Madrid). Spain; 10 Servicio de Geriatría. Hospital Universitario San Carlos. Departamento de Medicina. Facultad de Medicina. Universidad Complutense de Madrid. C/Profesor Martín Lagos s.n. 28040 Madrid. SPAIN

## Abstract

**Background:**

Disease management programmes (DMPs) have been shown to reduce hospital readmissions and mortality in adults with heart failure (HF), but their effectiveness in elderly patients or in those with major comorbidity is unknown. The Multicenter Randomised Trial of a Heart Failure Management Programme among Geriatric Patients (HF-Geriatrics) assesses the effectiveness of a DMP in elderly patients with HF and major comorbidity.

**Methods/Design:**

Clinical trial in 700 patients aged ≥ 75 years admitted with a primary diagnosis of HF in the acute care unit of eight geriatric services in Spain. Each patient should meet at least one of the following comorbidty criteria: Charlson index ≥ 3, dependence in ≥ 2 activities of daily living, treatment with ≥ 5 drugs, active treatment for ≥ 3 diseases, recent emergency hospitalization, severe visual or hearing loss, cognitive impairment, Parkinson's disease, diabetes mellitus, chronic obstructive pulmonary disease (COPD), anaemia, or constitutional syndrome. Half of the patients will be randomly assigned to a 1-year DMP led by a case manager and the other half to usual care. The DMP consists of an educational programme for patients and caregivers on the management of HF, COPD (knowledge of the disease, smoking cessation, immunizations, use of inhaled medication, recognition of exacerbations), diabetes (knowledge of the disease, symptoms of hyperglycaemia and hypoglycaemia, self-adjustment of insulin, foot care) and depression (knowledge of the disease, diagnosis and treatment). It also includes close monitoring of the symptoms of decompensation and optimisation of treatment compliance. The main outcome variables are quality of life, hospital readmissions, and overall mortality during a 12-month follow-up.

**Discussion:**

The physiological changes, lower life expectancy, comorbidity and low health literacy associated with aging may influence the effectiveness of DMPs in HF. The HF-Geriatrics study will provide direct evidence on the effect of a DMP in elderly patients with HF and high comorbidty, and will reduce the need to extrapolate the results of clinical trials in adults to elderly patients.

**Trial registration:**

(ClinicalTrials.gov number, NCT01076465).

## Background

Ninety percent of persons with incident heart failure (HF) are age 65 or over; moreover, the prevalence of HF increases with age, rising from 5-10% in persons aged 65-79 years to 10-20% in those 80 years or over [1]. In Spain, about 500,000 persons over the age of 60 suffer from HF [2]. HF is the most frequent reason for hospital admission and readmission [3] and is the third leading cause of cardiovascular death in the elderly [4]. Moreover, it represents about 2% of total health costs [5]. The number of hospitalisations for HF is expected to increase up to 50% in the next 20 years [6].

HF produces physical and cognitive impairment as well as reduced quality of life [7-9]. Annual mortality exceeds 50% in patients with New York Heart Association (NYHA) classes III and IV [10]. Advanced age, as well as low quality of life and poor social network - two variables which are not routinely assessed in patients - are all associated with a higher risk of hospitalisation and death in HF [11,12].

Treatments are available to modify the course of HF with left ventricular systolic dysfunction (LVSD) [13,14], but they have not managed to significantly reduce mortality and hospital readmission in routine clinical practice [15,16]. The prognosis for HF with preserved systolic function is similar to that of HF with depressed ejection fraction, but there is no evidence of effective treatments in the latter group, and clinical practice guidelines offer few specific recommendations for its treatment. The current therapeutic approach for these patients is symptomatic treatment, together with control of cardiovascular risk factors and heart rate [13,14].

### Management of HF

There is good evidence in patients with HF and high risk of hospitalisation that multidisciplinary strategies based on coordination and continuity of care can reduce mortality by 25%, hospital admissions for HF by 26-44%, and admissions for any cause by 19-27%, without increasing length of stay [17]. Thus, implementation of these disease management programmes (DMPs) is recommended in the major clinical practice guidelines [13,14]. In these DMPs a case manager provides patients and their caregivers with sufficient information and support to develop and comply with a plan to control HF. The case managers also reinforce compliance with treatment and detect early signs of HF decompensation.

In Spain, several DMPs have been evaluated in elderly HF patients. Morcillo et al. randomised 70 patients with severe HF and major systolic dysfunction (NYHA III or IV and left ventricular ejection fraction < 35%), previous hospitalisation for HF and mean age 79.1 years to "usual care" versus intervention by a case manager. At 6 months follow-up there were fewer deaths, emergency department visits and hospital readmissions in the intervention group [18]. However, the exclusion criteria were very strict, so that these patients may not be representative of many patients with HF. Atienza et al., in a randomised trial in 338 patients with a mean age of 68 years, showed that a DMP in tertiary hospitals can reduce readmissions for HF by 30% [19]. Lupón et al. obtained a 49% reduction in admission of patients with HF after 1 year of applying a HF programme (non-randomised before-and-after analysis) [20]. Brotons et al. recently reported a randomised home-based intervention study in 283 patients with a mean age of 76 years, 89% of whom had NYHA class III-IV HF. They observed a considerable reduction in the combined endpoint readmission or death at 1-year follow-up; however, only 25% of the patients initially evaluated could be included because the rest either did not meet the selection criteria or refused to participate [21].

### HF management programmes in patients with advanced age and major comorbidity

The mean age of patients included in studies that have evaluated the effectiveness of DMPs in HF is 73 years, which is substantially lower than that of patients hospitalised for HF in geriatric services [22]. Most clinical trials in HF have excluded elderly (75-85 years) and very elderly (> 85 years) patients with significant functional impairment, polymedication, physical comorbidity, and preserved ventricular function. This is important because 95% of elderly HF patients have non-cardiac conditions that make their clinical management difficult and lead to increased mortality [23]. Furthermore, the process and structure of DMPs in HF vary widely, therefore it is difficult to identify which specific interventions account for their benefits [23].

For all these reasons, we do not know the effectiveness of DMPs or of their main components in geriatric patients with HF. Specifically, it is unclear whether systematic assessment and intervention on psychosocial factors can improve these patients' vital prognosis and quality of life. It is particularly important to test whether interventions useful in younger HF patients are also useful in elderly patients with predominantly preserved systolic function and some level of comorbidity [24].

Finally, little information is available on the long-term outcomes of DMPs in HF. Although the effects are maintained up to 6 months [25], it has also been observed that interruption of DMPs is accompanied by loss of their effect [26]. Thus, future evaluations of DMPs should cover a minimum period of 1 year.

### Study objectives

The main objective of the HF-GERIATRICS study is to assess the effectiveness of a DMP in elderly patients with HF and major comorbidity over 12 months. Effectiveness will be based upon hospital readmission, health-related quality of life, and mortality from any cause.

The secondary objectives are to assess the impact of the DMP on some intermediate variables such as knowledge of the disease by patients and family members, and improved HF care, which includes compliance with recommendations on lifestyles and drug treatment. This study also aims to measure the impact of the DMP on length of hospital readmissions and to identify the clinical, demographic and psychosocial characteristics of patients who benefit most from the DMP.

## Methods/Design

### Design

The HF-GERIATRICS study is a trial in elderly patients who are admitted with a primary diagnosis of HF in acute care units of eight geriatric services in Spain. Patients are assigned randomly to a DMP (intervention group) or to receive usual care (control group). Randomisation is stratified by hospital and is conducted with concealment of the randomisation list. The two trial groups are followed for 12 months.

The project has been approved by the clinical research ethics committee of all participating hospitals (Comité de Etica y Ensayos Clínicos (CEIC) de Galicia, CEIC del Complejo Hospitalario de Caceres, CEIC del Hospital Universitario La Paz, CEIC del Complejo Universitario de Albacete, CEIC del Hospital Universitario de Guadalajara, CEIC del Hospital Central de Asturias, CEIC del Hospital Universitario de Getafe, CEIC del Hospital Universitario de San Carlos, Spain).

### Study participants

The study will include patients aged 75 years or over who are admitted with a primary diagnosis of HF according to the European Society of Cardiology and Framingham criteria [14,27]. Each patient must also meet at least one of the following comorbidity criteria: Charlson index ≥ 3 (regardless of age), dependence in ≥ 2 basic activities of daily living, prescription of ≥ 5 drugs, emergency hospitalisation in the last 3 months, active treatment for ≥ 3 diseases, limitations in daily life due to visual or hearing loss, cognitive impairment, Parkinson's disease, diabetes mellitus, chronic obstructive pulmonary disease (COPD), anaemia or constitutional syndrome whose causes have not been determined previously.

Exclusion criteria are terminal or rapidly fatal disease and life expectancy of less than 6 weeks, severe functional or cognitive impairment preventing patients from understanding their illness and lack of a caregiver who agrees to participate in the study, patients waiting for cardiac surgery, those who cannot be followed up (e.g., due to change of address), and institutionalised persons for whom a primary caregiver cannot be identified.

### Identification of participants, informed consent and randomisation

In each hospital a case manager with a degree in health sciences and special training in HF will identify and register cases admitted for HF each day by reviewing the diagnoses of those aged ≥ 75 years admitted in the acute care unit of the geriatric service. The case manager will then assess each patient and carry out the following activities:

i- Verify the study inclusion and exclusion criteria.

ii- Inform patients (verbally and in writing) and their caregivers (when considered necessary and with the patient's consent) of the characteristics of the DMP and confirm their agreement to participate by asking them to sign the informed consent document.

iii- Assign the patient to the intervention or control group. Randomisation will be conducted independently in each centre. Consecutively numbered envelopes, containing the group to which each patient should be assigned, will be used for this purpose. The envelope will be opened only at the time of patient assignment, to assure concealment of the randomisation list.

iv- Coordinate patient assessment, record the information in the case report form, and transcribe the information to a computerized database.

### Description of the intervention: Disease Management Programme

The DMP, which will be carried out by the case manager, has three main components: education of the patient and the main caregiver to improve disease knowledge and self-management, monitoring of the patient's clinical condition, and monitoring of treatment compliance.

a) DMP components to be conducted by the case manager during the hospital stay

i- Provision of information to patients and their caregivers or family members. An individual session will be held to teach the characteristics, causes and prognosis of HF. Emphasis will be given to recognition of symptoms of decompensation, precipitating factors, and treatment (drug knowledge and treatment compliance).

ii- Provision of written and graphic educational materials with the foregoing information.

iii- Health education on warning signs and symptoms and information on drugs for the most common comorbidities: diabetes, COPD and depression.

iv- Coordination of hospital discharge with ward nursing staff

v- Development of a follow-up plan. A date for an outpatient appointment will be set, and a telephone contact number will be provided to patients so they can ask questions or report any incidents.

b) Components of the DMP to be conducted by the case manager after hospital discharge

The case manager will devote 2 days/week to the clinical assessment of outpatients. The first contact with the patient will be by telephone, in the second week after hospital discharge: during this call, the case manager will verify the patient's knowledge of HF, reinforce the information provided during hospitalisation, and strengthen compliance with recommended lifestyles and pharmacological treatment. Control of minor symptoms of decompensation by use of diuretics will be emphasized. The first visit will take place at the hospital outpatient clinic 4 weeks after discharge. Future visits will be agreed according to the patient's clinical and treatment needs, but some minimum controls will be established at months 1, 2, 3, 6, 8 and 12 after discharge (table 1). The case manager will be provided with a cell phone to facilitate coordination with patients, caregivers and other health professionals. Messages left with the case manager will be answered within 24 hours.

**Table 1:** Design and data collection in the HF-Geriatrics study

TIME	CONTROL GROUP	INTERVENTION GROUP	WHO DOES IT
Admission	Review of inclusion and exclusion criteria Informed consent Randomisation **BASELINE VARIABLES**		Case manager
		
	ROUTINE MEDICAL MONITORING (will vary across centres)	Educational session Knowledge of disease Drugs Comorbidity Follow-up information Plan for discharge Questionnaires on self-care and disease knowledge for patient and caregiver	
		
2 weeks		Telephone contact Symptoms-signs Compliance Next appointment	Case manager
		
1 month		Consultation with case manager Educational reinforcement Drugs-Compliance Comorbidity Follow-up	Case manager
		
2 months		Consultation with case manager Similar to above	Case manager
		
3 months		Consultation with case manager Similar to above	Case manager
		
**6 months**		**Consultation with physician**	**Physician**
		
		Consultation with case manager Similar to above	Case manager
	
	**REGISTRY OF VARIABLES**		**Research team**

8 months		Consultation with case manager Similar to above	Case manager
		
**12 months**		**Consultation with physician**	**Physician**
		
		Consultation with case manager Similar to above	Case manager
	
	**REGISTRY OF VARIABLES**		**Research team**

The DMP will be supervised by the geriatricians on the research team, who will progressively delegate clinical functions to the case manager. A training programme for case managers will be conducted before the beginning of the trial. The DMP includes protocols for HF diagnosis and treatment will be implemented, based on the clinical guidelines of the European Society of Cardiology [14]. It also includes protocols to optimise the diagnosis and treatment of COPD, diabetes mellitus and depression. All cases of COPD are to be confirmed by spirometry, and patients and their caregivers will be trained on the use of inhaled drugs, smoking cessation, use of vaccines, and identification and management of exacerbations; in addition, treatment will be established in accordance with the Global Initiative for Chronic Obstructive Lung Disease (GOLD) recommendations. Patients with diabetes mellitus will receive an educational intervention on the symptoms of hypoglycaemia and hyperglycaemia, foot care, and, for patients treated with insulin, on the proper technique for its administration and self-monitoring of capillary blood glucose. In cases of depression, a clinical assessment of the diagnosis will be made if the score on the 15-item Yessavage test is > 5, and appropriate treatment will be initiated.

At outpatients clinic visits and telephone contacts, case managers will identify warning signs and symptoms of clinical deterioration from records of blood pressure and heart rate, changes in body weight, night-time cough, degree of dyspnea, decreased physical activity, and biomarkers such as renal function, natriuretic peptides and haemoglobin saturation by pulse oximetry.

For patients in the control group, the same intermediate and outcome variables will be recorded as in the intervention group, but the previously described educational activities and monitoring by the case manager will not be carried out. Follow-up will begin with the index admission and ends 12 months after discharge or in case of the patient's death.

### Study variables

In addition to the inclusion and exclusion criteria, four types of variables will be collected: psychosocial, biomedical, treatment prescribed, and intermediate and final outcome variables (table 2). Episodes of hospital emergency care will also be recorded. The intermediate outcome variables are knowledge of the nature and management of HF and of comorbidity, and compliance with recommendations on lifestyle and drug treatment. The final outcome variables are health-related quality of life (assessed using the Minnesota Living with Heart Failure Questionnaire [MLWHFQ]), hospital readmissions, and total mortality. The outcome variables are measured at 6 and 12 months after the DMP begins. The information is collected through interview with the patient or primary caregiver and review of the clinical record.

**Table 2:** Data collected in the study

Type of data	Variables
Definition of inclusion criteria	Hospitalisation.- hospital stay at least one night. Heart failure.- Framingham and European Society of Cardiology criteria Comorbidity.- Charlson index and other (see text) [34]
Psychosocial variables	*Sex ^#^Dependence in ADLs and IADLs.- Index of Katz [35] and Lawton [35,36] ^#^Quality of life.- Minnesota Living With Heart Failure Questionnaire [37] ^#^Depression.- 15-item Yessavage depression scale [38] ^#^Cognitive function.- Lobo cognitive miniexam [39] ^#^Health literacy.- SHALSA questionnaire[40] ^#^Knowledge of disease [33] ^#^Knowledge of treatment and self-care measures [41] *Housing conditions [42] *Social support.- OARS questionnaire [43] ^#^Treatment compliance.- interview and Morisky-Green test.
Biomedical variables	*Age *Pneumococcal and influenza vaccinations *Cardiovascular risk factors.- hypertension, smoking, diabetes, hypercholesterolemia and obesity. *Factors leading to admission *Length of hospital stay *Aetiology of heart failure ^#^Functional class (NYHA) *Ejection fraction.- transthoracic echocardiography ^#^Blood sodium, NTpBNP, creatinine and glucose ^#^Blood pressure, height and weight (body mass index)
Treatment	^#^Total number of drugs ^#^Drugs.- loop and potassium-sparing diuretics, angiotensin converting enzyme inhibitors, angiotensin receptor blockers, beta-blockers, antiplatelets, anticoagulants, statins ^#^Adverse effects associated with treatment
Number of emergency department visits	^#^To be detected by review of the computerized clinical history or by the hospital admission records, counting all episodes after the date of discharge of the index admission. The number of these visits directly related with heart failure will be recorded.
Intermediate outcome variables	^#^Knowledge of disease ^#^Self-care measures
Final outcome variables	^#^Readmission.- all hospitalisation episodes of more than 24 hours following the date of discharge of the index admission. The following variables will be recorded: number and proportion of patients who are readmitted, number of readmissions per patient, and length of hospital stay during readmissions. ^#^Death.- To be determined by follow-up of each patient. This will be documented in the clinical history and the mortality registries. ^#^Quality of life.- Measured by the Minnesota Living With Heart Failure Questionnaire

ADLs: Basic activities of daily living; IADLs: Instrumental activities of daily living; * Variables to be registered only during the index admission; # Variables to be registered at the index admission and at 6 and 12 months.

### Study size

In previous studies we observed that about 30% of HF patients are readmitted every 6 months [11]. These studies were conducted in hospitals and with patient's inclusion criteria different from those in the HF-Geriatrics, and within patients with less use of effective drug treatments than at present. Accordingly, under the conservative assumption that the annual frequency of readmission will be 40%, and that the frequency of readmission is similar in all study hospitals, 700 patients will need to be recruited (350 in each group) to detect, with an alpha error of 5% and a statistical power of 80%, a 20% reduction in hospital admission. This analysis assumes, as can be expected, that no patients will be lost to follow-up. A total of 700 patients also allows to detect, with an alpha error of 5% and 80% power, differences of 10 points (clinically relevant) in the MLWHFQ, assuming that patients in the control group have a mean score of 50 points on this questionnaire [11].

### Statistical analysis

We will first verify the effectiveness of randomisation. The calculated sample size should be sufficient to assure an even balance of the predictors of the outcome variables between the intervention and control groups.

Second, we will estimate the time to readmission or death for each patient, and will obtain curves for time free of such events (Kaplan-Meier method) in the intervention and control groups. These curves will be compared using univariate methods. The effectiveness of DMP will be summarized with hazard ratios (and their 95% confidence intervals) for readmission and mortality obtained by Cox regression, using the hospital as stratification variable. Finally, if the DMP is shown to be effective, we will estimate the number of patients needed to treat (NNT) to prevent one outcome event.

For health-related quality of life, the MLWHFQ score at baseline, 6 months and 12 months will be calculated for each group in the trial. The effect of the DMP on quality of life will be estimated as the difference between groups in the change in MLWHFQ scores during the study. These differences will be accompanied by their respective 95% confidence intervals.

The analyses of the effect of DMP will be performed according to the intention-to-treat principle.

## Discussion

To our knowledge, this is the first evaluation of a DMP in HF targeting elderly patients with major comorbidity. Elderly patients should be included in evaluations of DMPs because they have specific clinical characteristics. First, the elderly have a lower life expectancy than younger adults; thus, the interventions must be successful in the short or medium term. In addition, their health status is more heterogeneous, and the expectations and preferences of these patients are different from what they were at younger ages. Second, the effect of interventions on outcomes like quality of life and functional status should receive as much attention as the "hard" endpoints traditionally studied (e.g., hospital readmission, mortality, etc.). Third, the prognostic importance of comorbidity in the elderly is similar to or even greater than that of HF itself. Thus, control of comorbidity should be an important target of DMPs. In other words, DMPs for HF in the elderly should "look beyond the heart" [28]. Designing interventions for patients with comorbidity is complex since we must select those with the greatest possibility of success to avoid overburdening patients with treatments of doubtful therapeutic value. For this reason, the HF-Geriatrics study has selected comorbidities for which there is good evidence of the benefit of the intervention [29-32].

The main strengths of this study are random assignment to the intervention, concealment of the randomisation list, intention-to-treat analysis, and the very nature of the DMP designed. This DMP follows the recommendations of the major scientific societies in this field (13,14) and has been refined through a literature search for successful interventions. In addition, the intervention will be adapted to the educational level and visual limitations of most patients with the following measures: a) selection of a limited number of self-care skills to manage HF, COPD, diabetes mellitus and depression, to identify the warning signs of decompensation, and to comply with treatment [33]; b) implementation of only two brief educational sessions, the first during hospitalisation and the second on the day of discharge, as well as a telephone call 15 days after discharge; c) development of printed educational materials with brief text and large print, avoiding technical terms and with numerous explanatory pictures, to be given to patients in the intervention group on the day of hospital discharge; d) provision of a telephone contact number to patients so they can ask questions or resolve any issues they may have.

We plan to conduct specific training and certification of the case managers in the coordinating centre and to develop protocols for each of their activities. This ensures that the same intervention will be applied similarly in all participating centres and that, if the efficacy of the DMP is shown, it can easily be expanded to other health care settings.

The main challenges of the project are recruitment of study participants, avoidance of contamination of the intervention between the two trial groups, and minimization of losses to follow-up. To ensure sufficient recruitment, we have reviewed the basic hospital discharge dataset for each participating hospital, and have found that they each admit at least 100 patients with the characteristics needed for the project every 6 months. To avoid contamination, the case manager will conduct the DMP in a separate consultation, at a time of day other than when patients usually receive care. Finally, to minimize losses to follow-up, the intervention will be brief and will include only a small number of hospital visits. In addition, patient access to the case manager will be facilitated (by telephone or in person) so that any questions or problems that arise during the intervention can be addressed.

## Competing interests

The authors declare that they have no competing interests.

## Authors' contributions

CRP is the scientific coordinator of the study. CRP and FRA drafted the manuscript. All authors contributed to the study concept and design, revised the manuscript for important intellectual concept, and approved the final manuscript.

## Pre-publication history

The pre-publication history for this paper can be accessed here:

http://www.biomedcentral.com/1471-2458/11/627/prepub
